# Functional *KRAS* mutations and a potential role for PI3K/AKT activation in Wilms tumors

**DOI:** 10.1002/1878-0261.12044

**Published:** 2017-03-15

**Authors:** Dina Polosukhina, Harold D. Love, Hernan Correa, Zengliu Su, Kimberly B. Dahlman, William Pao, Harold L. Moses, Carlos L. Arteaga, Harold N. Lovvorn, Roy Zent, Peter E. Clark

**Affiliations:** ^1^ Department of Urologic Surgery Vanderbilt University Medical Center Nashville TN USA; ^2^ Department of Pathology, Microbiology & Immunology Vanderbilt University Medical Center Nashville TN USA; ^3^ Vanderbilt‐Ingram Cancer Center Nashville TN USA; ^4^ Department of Cancer Biology Vanderbilt University Medical Center Nashville TN USA; ^5^ Department of Medicine (Hematology‐Oncology) Vanderbilt University Medical Center Nashville TN USA; ^6^ Department of Pediatric Surgery Vanderbilt University Medical Center Nashville TN USA; ^7^ Department of Medicine, Nephrology & Cancer Biology Division Vanderbilt University Medical Center Nashville TN USA

**Keywords:** AKT, KRAS, Wilms tumor, β‐catenin

## Abstract

Wilms tumor (WT) is the most common renal neoplasm of childhood and affects 1 in 10 000 children aged less than 15 years. These embryonal tumors are thought to arise from primitive nephrogenic rests that derive from the metanephric mesenchyme during kidney development and are characterized partly by increased Wnt/β‐catenin signaling. We previously showed that coordinate activation of Ras and β‐catenin accelerates the growth and metastatic progression of a murine WT model. Here, we show that activating *KRAS* mutations can be found in human WT. In addition, high levels of phosphorylated AKT are present in the majority of WT. We further show in a mouse model and in renal epithelial cells that Ras cooperates with β‐catenin to drive metastatic disease progression and promotes *in vitro* tumor cell growth, migration, and colony formation in soft agar. Cellular transformation and metastatic disease progression of WT cells are in part dependent on PI3K/AKT activation and are inhibited via pharmacological inhibition of this pathway. Our studies suggest both *KRAS* mutations and AKT activation are present in WT and may represent novel therapeutic targets for this disease.

**Abbreviations**
BCS: body condition score
IHC: immunohistochemistry
MAPK: MEK/ERK mitogen‐activated protein kinase
RTKs: receptor tyrosine kinases
TMA: tissue microarray
WT1: Wilms tumor 1
WT: Wilms tumor
WTX: Wilms tumor gene found on chromosome X

## 1 Introduction

Wilms tumor (WT) is the fourth most common malignancy of childhood and the most common renal neoplasm (Grovas *et al*., 1997; Gurney *et al*., 1995). The majority of affected children are cured with modern multimodal therapy (Dome *et al*., 2006; Metzger and Dome, 2005; Sonn and Shortliffe, 2008; Tournade *et al*., 2001; Varan, 2008); however, these therapies are associated with significant short‐ and long‐term morbidity (Green *et al*., 2001, 2002; Jones *et al*., 2008; Taylor *et al*., 2008). Additionally, there remains a substantial proportion of patients who relapse, of whom up to 50% may die of disease progression depending on their risk group (Dome *et al*., 2006, 2015). A principal challenge in WT is identifying novel, target‐specific drugs that lower treatment morbidity while maintaining treatment efficacy and improving tumor responses. Such therapeutic advances rely on a deep understanding of the mechanisms underlying WT disease progression.

Wilms tumor are triphasic, embryonal tumors that arise from primitive nephrogenic rests derived from the metanephric mesenchyme during renal development. The genetic aberrations underlying this process are varied and include inactivating mutations of Wilms tumor 1 (*WT1*) (Huff, 1998; Ruteshouser *et al*., 2008), Wilms tumor gene found on chromosome X (*WTX*) (Fukuzawa *et al*., 2010; Perotti *et al*., 2008; Rivera *et al*., 2007), and stabilizing/activating mutations of β‐catenin (*CTNNB1*) (Koesters *et al*., 1999; Maiti *et al*., 2000). While the precise mechanisms driving Wilms tumorigenesis are not clear, each shares an association with increased Wnt/β‐catenin signaling (Kim *et al*., 2009, 2010; Koesters *et al*., 1999; Major *et al*., 2007). However, aberrant canonical WNT signaling alone is a weak inducer of WT formation and does not appear to promote disease progression by itself (Clark *et al*., 2011).

The Ras family is a group of membrane‐bound GTPase proteins that regulate numerous cellular processes by activating signaling pathways such as the MEK/ERK mitogen‐activated protein kinase (MAPK) and PI3K‐AKT pathways. Constitutively active KRAS has been implicated in numerous human cancers, including the pancreas, lung, brain, and colon, due to its ability to activate downstream RAF/MEK/ERK and PI3K/AKT. One mechanism whereby these activated MEK/ERK and PI3K/AKT pathways induce oncogenesis is by regulating β‐catenin activation, as documented in breast cancer (Faivre and Lange, 2007; Jang *et al*., 2006), melanoma (Delmas *et al*., 2007), prostate cancer (Pearson *et al*., 2009), and colon cancer (Chakladar *et al*., 2005; Janssen *et al*., 2006; Li *et al*., 2005; Ramsay *et al*., 2005; Sansom *et al*., 2006; Yeang *et al*., 2008).

We previously showed that coordinate activation of β‐catenin and Ras in mouse kidney epithelium accelerates the development and metastatic progression of primitive renal epithelial tumors that strongly resemble human WT both genetically and histologically (Clark *et al*., 2011; Yi *et al*., 2015). This model of metastatic WT is characterized by significant intratumoral activation of AKT. Here, we show that human WT and our murine model harbor identical *KRAS*‐activating mutations. Further, the majority of human WT exhibit high levels of AKT activation. Utilizing a novel murine WT cell line, we show that Ras and β‐catenin cooperate to accelerate tumor cell growth, migration, and colony formation *in vitro* and growth and metastatic disease progression of orthotopic grafts. Cellular transformation and metastatic progression are in part PI3K/AKT dependent and are inhibited through pharmacological inhibition of PI3K/AKT using the pan‐PI3K small‐molecule antagonist, BKM120 (buparlisib), currently in late clinical development (Ando *et al*., 2014; Bendell *et al*., 2012; Hyman *et al*., 2015; Rodon *et al*., 2014) Thus, our studies demonstrate WT can harbor *KRAS* mutations and that targeting PI3K/AKT activation in WT may be a viable new strategy in treating these tumors.

## 2 Materials and methods

### 2.1 Mice

Mice with a conditional activating mutation of *Ctnnb1,* in which exon 3 is flanked by lox sites (Catnb^lox(ex3)^), were a kind gift from Makoto M. Taketo (Harada *et al*., 1999). Mice with a conditional activating mutation of *Kras* (*LSL‐Kras* ^G12D^) were obtained from Tyler Jacks (Massachusetts Institute of Technology) (Jackson *et al*., 2001). These strains were crossed to obtain genotypes *Kras* ^+/G12D^/*Catnb* ^+/+^, *Kras* ^+/+^/*Catnb* ^+/lox(ex3)^, and *Kras* ^+/G12D^/*Catnb* ^+/lox(ex3)^. Six mice of each genotype were euthanized at 6 weeks of age in order to generate the floxed renal epithelial cell lines described subsequently. All mice were bred and housed under an Institutional Animal Care and Use Committee‐approved protocol.

### 2.2 Antibodies and reagents

Antibodies used for immunohistochemistry (IHC) and/or western blot were as follows: S‐100 (Dako, Santa Clara, CA, USA), Pax‐2 (Covance, Princeton, NJ, USA), Pax‐8 (Proteintech Group, Chicago, IL, USA), actin (Sigma‐Aldrich, St Louis, MO, USA), WT‐1 (Leica Microsystems, Buffalo Grove, IL, USA), CD56/NCAM (Invitrogen, Waltham, MA, USA), SALL4 (Abnova, Taipei Taiwan), and total and p‐AKT, PARP, cleaved caspase 3 (Cell Signaling Technology, Boston, MA, USA). The pan‐PI3K kinase inhibitors utilized were LY294002 (EMD biosciences, San Diego, CA, USA) and BKM120 (Active Biochem, Maplewood, NJ, USA).

### 2.3 SNaPShot mutational profiling assay

DNA was extracted from 10 micron sections of formalin‐fixed, paraffin‐embedded tumor blocks (*n* = 19 deidentified clinical WT specimens), and specific mutations in *KRAS*,* BRAF*,* AKT*,* PIK3CA*,* SMAD4*,* PTEN*, and *NRAS* were queried using a SNaPShot mutation profiling approach. The SNaPShot mutational profiling method is characterized by multiplexed PCR and multiplexed single‐base primer extension, followed by capillary electrophoresis (Dias‐Santagata *et al*., 2010; Lovly *et al*., 2012; Su *et al*., 2011). The current assay was designed to detect 62 unique point mutations in these seven genes (Table S1). Briefly, PCR primers were pooled to amplify the target DNA, and PCR was performed using the following conditions: 95 °C (8 min), followed by 40 cycles of 95 °C (20 s), 58 °C (30 s), and 72 °C (1 min), and then a final extension of 72 °C (3 min) (Table S2). Next, PAGE‐purified primers were pooled together, and multiplex single‐base extension reactions were performed on Exo‐SAP‐it treated (USB) PCR products using the following conditions: 96 °C (30 s), followed by 35 cycles of 96 °C (10 s), 50 °C (5 s), and 60 °C (30 s) (Table S3). Extension products were applied to capillary electrophoresis in an ABI 3730 analyzer, and the data were interpreted using abi genemapper software (version 4.0; Waltham, MA USA). Human male genomic DNA (Promega, Madison, WI, USA) was used as a wild‐type control. Spiking primers were mixed to create a pan‐positive control mix for the assay (Table S4).

### 2.4 Sanger DNA sequencing

Ten‐micron sections of formalin‐fixed, paraffin‐embedded tumor blocks were shipped to GENEWIZ^®^ (South Plainfield, NJ, USA) which performed Sanger sequencing on a fee‐for‐service basis using standardized techniques.

### 2.5 Human WT tissue microarray

The construction of our WT tissue microarray (TMA) has been published previously (Murphy *et al*., 2015; Pierce *et al*., 2014). In brief, we prospectively collected and archived in our IRB‐approved laboratory embryonal tumor repository formalin‐fixed, paraffin‐embedded, renal tumor and adjacent kidney specimens from 21 consecutive childhood WT. From this, we created a TMA comprising 72 total punches (~ 1 mm in diameter each) derived from these patients' specimens. Serial 5‐μm sections of these two TMAs were included for the IHC analysis, which was concentrated on the 21 WT specimens.

### 2.6 Generation of renal epithelial cell lines

Six C57BL/6 mice with genotypes *Kras* ^+/G12D^/*Catnb* ^+/+^, *Kras* ^+/+^/*Catnb* ^+/lox(ex3)^, or *Kras* ^+/G12D^/*Catnb* ^+/lox(ex3)^ were euthanized at 6 weeks of age, and the renal papillae tissue was harvested, manually disrupted, and maintained in media under sterile conditions. Primary cultures of renal epithelial cells were subsequently isolated and immortalized with SV40 large T antigen, as previously described (Chen *et al*., 2004; Wang *et al*., 1999). Recombination was induced *in vitro* by adding adenoviral Cre‐recombinase. The nonrecombined floxed populations were retained as controls. Recombination was confirmed by PCR using the following primer pairs: for *Kras*, 5′CAGTGCAGTTTTGACACCAGCT3′ and 5′GCATAGTACGCTATACCCTGTGGA3′, and for *Ctnnb1*, 5′TGAAGCTCAGCGCACAGCTGCTGTG3′ and 5′ACGTGTGGCAAGTTCCGCGTCATCC3′. The cycling sequence was as follows: 94 °C for 30 s, 65 °C for 1 min, 72 °C for 90 s, for 39 cycles. The resulting recombinant cell lines had an activating mutation of *Kras*,* Ctnnb1*, or both and are referred to as Kras, Catnb, and Kras/Catnb cells. Cells were maintained in DMEM with 5% FBS under standard culture conditions and used for subsequent experiments.

### 2.7 Histology and immunohistochemistry

Murine kidneys were harvested, fixed in 10% buffered formalin, processed, and paraffin‐embedded. Sections were either stained with hematoxylin and eosin (H&E) or subjected to IHC. For IHC, the slides were incubated with primary antibodies and then exposed to biotinylated secondary antibody followed by incubation with an ABC–HRP complex (Vector Laboratories) and then with liquid 3,3′‐diaminobenzidine tetrahydrochloride (DAB) (DAKO liquid DAB + substrate chromogen system, Carpinteria, CA). Stained sections were photographed and processed using a Zeiss AX10 Imager.M1 microscope and axiovision release 4.6 software (Gottingen, Germany). The intensity of phosphorylated Akt was assessed using a semiquantitative 3‐point scale (0–3+) and the proportion of cells staining called by a dedicated pediatric pathologist (HC).

### 2.8 Cell viability assay

Cell viability was determined using the MTS method (Promega) using the manufacturer's protocol. In brief, cells were seeded in 96‐well culture plate, grown overnight, and treated as indicated. MTS/PMS solution was added for 1 h and absorbance at 490 nm measured. All experiments were completed in triplicate, and the results are provided as the mean ± the standard error.

### 2.9 Tritiated thymidine incorporation

Cells were seeded onto 35‐mm dishes and treated as indicated. Tritiated thymidine was added, and the cells were incubated for 2 h. The media were removed, and the cells were incubated with 10% TCA solution, washed twice, and incubated with 0.2 N NaOH. Aliquots were combined with scintillation fluid and counted on a scintillation counter. All experiments were completed in triplicate.

### 2.10 Cellular migration/wound healing assay

Cells were grown in 6‐well plates to 100% confluence and pretreated with reduced serum (1% FBS) medium overnight. Media were removed and several parallel scratch lines (wounds) were made with sterile 200 μL pipette tip. Dislodged cells and debris were gently removed by washing with PBS and serum‐reduced media with or without inhibitors added. Baseline images of the same spots (at least 4 per well) were captured immediately and 6, 16, 24, 48 h after scratch. The distance between wound borders was measured using cellsens software (Olympus Corporation) as average of 15 parallel lines connecting cells across the wound. The average ± SE difference was then calculated. Each experiment was repeated in triplicate.

### 2.11 Cellular invasion assay

BD BioCoat™ Matrigel™ Invasion chambers (cat #354480, BD Biosciences, Bedford, MA, USA) were utilized according to the manufacturer's protocol. After warming and rehydration, 10^5^ cells were seeded with inhibitors or vehicle in serum‐free cell culture medium in the upper chamber/inserts, and full serum media with the same inhibitor were placed into the lower chamber/wells and incubated for 24 h. Inserts were then removed and fixed in 10% neutral buffered formalin and stained with Mayer's hematoxylin. Cells on the inner aspect of the insert were removed with a cotton swab, while cells on the outer membrane were counted by cutting out the insert and mounting them on a slide with a coverslip and allowed to dry overnight. Ten nonoverlapping pictures were captured for each membrane using cellsens life science imaging Software (Olympus Corporation). Cells were then counted using the same software, and the mean ± SE was calculated. Each experiment was repeated in triplicate.

### 2.12 Colony formation in soft agar

Six‐well plates were coated with a 1 : 1 mix of 1.6% sea plaque agarose (Cambrex Bio Science, Rockland, ME, USA) and 2x cell culture medium (with all additives and 2x serum) and allowed to solidify. A mix of 2x cell culture medium, sea plaque agarose, and 5000 cells with inhibitors or vehicle in 1x cell culture medium (ratio 1 : 1 : 2 by volume) was plated above the soft agar coat, allowed to solidify, and incubated at 37 °C for 4 weeks. The total number, size, and density of colonies were captured using the GelCount™ system (Oxford Optronix, Abingdon, UK) that includes the digital image capture and analysis software. Each experiment was repeated in triplicate.

### 2.13 Immunoblotting

Cells were washed and dissolved in lysis buffer (made fresh from a 6x stock solution of 2 m Tris/HCl pH 6.8, 20% SDS, glycerol, and protease inhibitors) and sonicated. Cell lysates were cleared by centrifugation. Protein concentration was determined using the Bio‐Rad protein assay and then subjected to SDS/PAGE, transferred to Immobilon‐P transfer membranes (Millipore Corporation, Billerica, MA, USA), and subjected to immunoblot analysis utilizing standard methods using the antibodies listed previously.

### 2.14 Orthotopic xenografts

For subrenal orthotopic grafting, 10^5^ cells were used per graft. Cells were trypsinized and pelleted and then resuspended in 50 μL of neutralized rat tail collagen, as described previously (Hallowes *et al*., 1980). The gels were allowed to set at 37 °C for 15 min and were then covered with growth medium (DMEM/F12, 5% FBS). Two collagen gels were then grafted beneath the left renal capsule of adult female athymic mice (Hsd:Athymic Nude‐Foxn1nu, Harlan Laboratories, Madison, WI, USA). For experiments comparing Kras, Catnb, and Kras/Catnb cells, animals were maintained and assessed utilizing a previously described body score index method (Ullman‐Cullere and Foltz, 1999). This method gives guidelines on assessing animal health using a body condition score (BCS), with 5 and 4 reflecting overweight mice and 3 reflecting mice that are in optimal condition. Mice that met these general criteria were observed on an ongoing basis for up to 1 year. Mice warranting a BCS of 2 (thin with prominent bones) or 1 (advanced muscle wasting) or that developed palpable masses in the flank were euthanized and the kidneys, liver, and lungs harvested to assess tumor size and metastases.

For the treatment experiments, grafts were allowed to establish for 14 days before starting the drug treatment. BKM120 (Active Biochem) was first dissolved in 1/10th volume of 1‐methyl‐2‐pyrrolidinone (NMP, Sigma‐Aldrich), and then diluted with 9/10th volume of PEG3000 (Sigma‐Aldrich) to a final concentration of 9 mg·mL^−1^. The BKM120 solution was then administered via oral gavage at a dose of 60 mg·kg^−1^·day^−1^, three times per week, for 4 weeks. Control mice received the NMP + PEG3000 vehicle. Mice were sacrificed 6 weeks after grafting (4 weeks of therapy), and kidneys and lungs were harvested to assess tumor growth and metastases. For each mouse, both the grafted and contralateral nongrafted control kidneys were weighed, and the tumor weight was expressed as the total weight of the grafted kidney normalized to its contralateral control. Lung metastases were manually counted after H&E staining using three serial sections at two different depths within the lung tissue (six sections for each lung per mouse); cellular origin of the tumor‐grafted cells was confirmed by IHC for SV40 large T antigen.

### 2.15 Statistical analysis

Descriptive statistics were expressed as the mean ± SE. Proportional differences were compared using contingency tables and Fisher's exact test. Comparison of continuous variables was made with Mann–Whitney test or one‐way ANOVA and Kruskal–Wallis test. All tests were completed using PRISM 5.0d^©^ (graphpad Software, Inc., La Jolla, CA, USA).

## 3 Results

### 3.1 *KRAS* mutations and increased AKT activation are present in human WT

**Figure 1:**
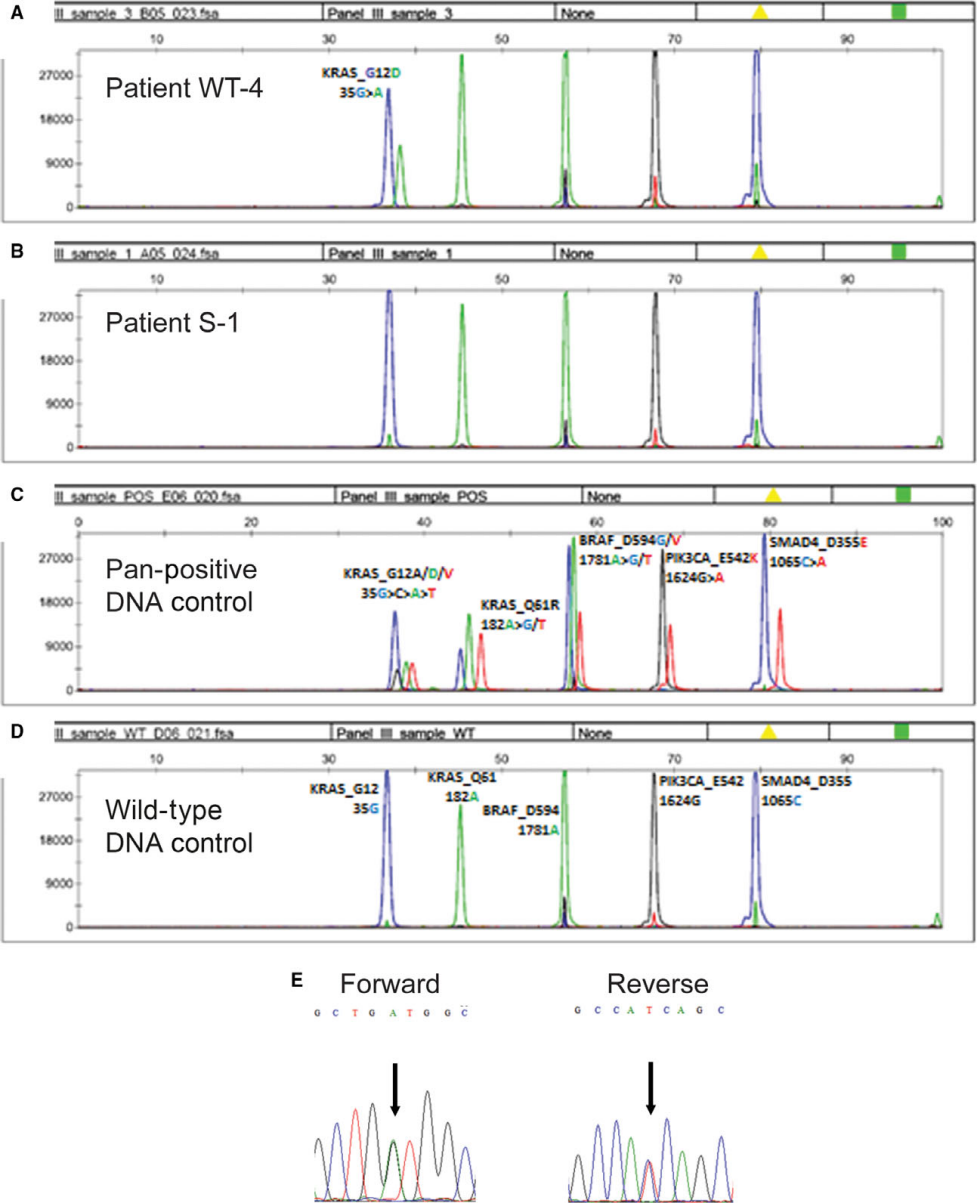
*KRAS*^G^ ^12D^ mutation in human WT: DNA was extracted from formalin‐fixed, paraffin‐embedded blocks of tumor tissue from 19 human WT and screened for mutations in a panel of genes using a multiplex PCR, multiplex primer extension, and capillary electrophoresis (SNaPShot method) screen. This was designed to screen for 62 unique mutations across seven genes (see Table S1). Shown is the result from a patient with a *KRAS*^G^ ^12D^ mutation (A), a patient with no mutation (B), as well as a pan‐positive (C) and wild‐type control (D). Point mutation in this patient was subsequently confirmed using Sanger sequencing (E).

**Figure 2:**
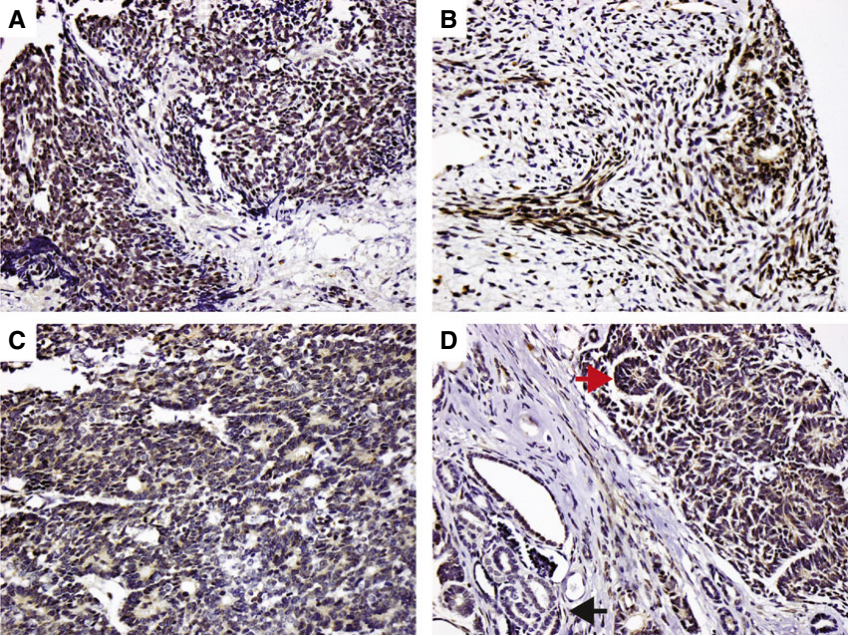
High levels of p‐AKT by immunohistochemistry in human WT: A tissue microarray of 72 tissue cores from 21 patients with WT stained by immunohistochemistry for p‐AKT. Staining intensity was scored for both intensity (0–3+) and by the proportion of cell staining. Of 21 patients, 15 (71%) demonstrated at least 2+ staining in 50% or more of the tumor. Shown are representative images at 20X power of WT showing 3+ staining in blastemal (A), stromal (B), and epithelial histologic elements (C). In the small number of areas where both were present in the same core (D), p‐AKT staining was higher in areas harboring primitive (red arrow) compared to more mature (black arrow) histologic epithelial elements. The magnification shown is 20×.

We previously reported that coordinate activation of KRAS and β‐catenin in murine kidneys causes the formation of primitive renal epithelial neoplasms that are histologically consistent with epithelial predominant WT and that are characterized by excessive ERK and AKT activation (Clark *et al*., 2011; Yi *et al*., 2015). To investigate whether *KRAS* mutations are present in human WT, we profiled 19 human WT specimens using a multiplex PCR, multiplex primer extension, and capillary electrophoresis (SNaPShot method) screen (Dias‐Santagata *et al*., 2010; Lovly *et al*., 2012; Su *et al*., 2011). A somatic *KRAS* ^G12D^ mutation, identical to that used in our transgenic mouse model of metastatic WT, was identified in one (5.3%) patient (Fig. 1A–D). Sanger sequencing confirmed the presence of this mutation (Fig. 1E). This patient was one of only two in the cohort who had a predominantly primitive epithelial tumor, the same histology seen in our murine model of metastatic WT (Clark *et al*., 2011). RAS can activate both MAPK/ERK and PI3K/AKT but only ERK has been shown to be activated in human WT (Hu *et al*., 2011). We therefore defined the prevalence of PI3K/AKT activation in a tissue microarray containing 72 cores from 21 human WT representing a range of histologies. The intensity of phosphorylated Akt was assessed using a semiquantitative 3‐point scale (0‐3+) and the proportion of cells staining called by a dedicated pediatric pathologist (HC); 15 (71%) of 21 patients demonstrated at least 2+ staining in 50% or more of the tumor cells (see Fig. 2A–C), and it was present in blastemal (panel 2A), stromal (panel 2B), and epithelial elements (panel 2C). Furthermore, in the small number of areas in which both mature and more primitive renal tubules were visible on the same core, the primitive renal epithelial elements demonstrated higher levels of phosphorylated AKT than the more mature appearing renal tubules (panel 2D). These data demonstrate that activating RAS mutations are found in human WT and most WT demonstrate activation of PI3K/AKT, suggesting a role for this pathway in pathogenesis of this disease.

### 3.2 Ras increases renal transformation through accelerated cell turnover and cellular migration

Based on the high degree of Akt activation in human WT, we sought to define the role of this pathway in the development and progression of this cancer. We therefore created a series of murine renal epithelial cell lines harboring either the *Kras* ^G12D^‐ or *Catnb* ^∆ex3^‐activating mutations or a combination of both (Harada *et al*., 1999; Jackson *et al*., 2001; Pozzi *et al*., 2006). Mice harboring one or both of the floxed alleles (*Catnb* ^lox(ex3)^, *LSL‐Kras* ^G12D^, or both) were euthanized at 6 weeks of age and their kidneys harvested. Renal epithelial tissue was grown out as a primary culture after immortalization with SV40 large T antigen. Recombination was induced *in vitro* using Cre‐recombinase adenovirus and the recombination confirmed by PCR (Fig. S1). The immortalized floxed parental cell lines were maintained as controls.

**Figure 3:**
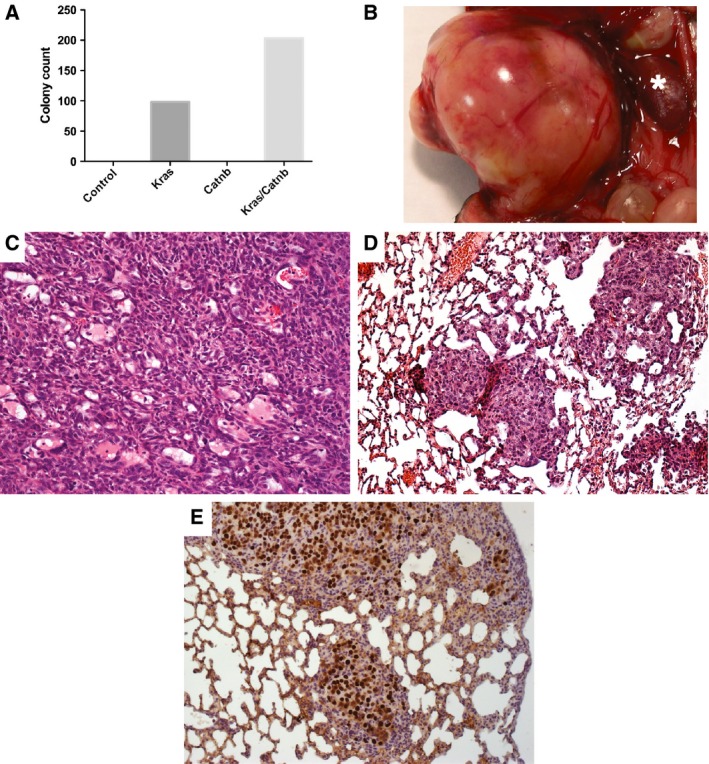
Kras/Catnb renal epithelial cells harboring activating mutations in *Kras* and *Ctnnb1* have increased cellular transformation and metastatic potential: (A) Colony formation in soft agar showing the highest number of colonies from Kras/Catnb cells when compared to Kras, Catnb, and control cells after 4 weeks. Experiment was completed in triplicate, representative results from one experiment shown here. (B) Kras, Catnb, or Kras/Catnb cells were implanted orthotopically under the right renal capsule, while control cells without recombination were grafted in the opposite left kidney. Mice were monitored for up to 1 year and then euthanized. Mice were euthanized earlier for poor health or for palpable tumors detected in the flank. By 10 weeks, all seven Kras/Catnb‐engrafted kidneys developed large tumors. Shown is a representative picture at necropsy (B) of a large tumor virtually replacing the right kidney, while the left kidney (*) grafted with control cells had no tumor growth. The H&E staining of the tumors from the graft is shown (C). By contrast, only one of six Kras‐engrafted kidneys developed a tumor by 8 weeks and one Catnb cell‐engrafted kidney developed a small tumor at 1 year. Five (71%) of seven mice engrafted with Kras/Catnb cells developed metastases in the lung (D), while no metastases were noted in Kras‐ or Catnb‐engrafted mice. Origin of the metastatic cells from the Kras/Catnb renal graft was confirmed by IHC staining for SV40 large T antigen in the lung metastases (E).

We initially tested whether the resultant cell lines, termed Kras, Catnb, and Kras/Catnb, would recapitulate the findings from our transgenic mouse model by comparing their ability to form colonies in soft agar. Kras/Catnb cells formed the most colonies, while Catnb or control cells formed no colonies (Fig. 3A). Colony formation was noted in cells with only Kras activation, although this mutation did not induce renal tumors in transgenic mice (Clark *et al*., 2011). To define the oncogenic potential of these renal epithelial cells *in vivo,* we grafted Kras, Catnb, or Kras/Catnb cells in the subrenal capsule of nude mice. By 10 weeks, all seven mice with Kras/Catnb cells formed large, invasive tumors in the renal capsule (see Fig. 3B,C), and there was evidence of lung metastases in five of seven mice (71%, Fig. 3D). The origin of the lung metastases was confirmed by staining for large T antigen (see Fig. 3E). Of the six mice grafted with Kras cells, only one formed a nonmetastatic tumor at 8 weeks, while the remainder did not form tumors even after 1 year of follow‐up. Similarly, only one of the six mice grafted with Catnb cells formed a small tumor at 1 year, and there was also no evidence of metastases. No kidneys grafted with control cells had any evidence for tumors or metastases (data not shown). Thus, simultaneous activation of Ras and β‐catenin in renal epithelial cells promotes cellular transformation, orthotopic tumor growth, and metastatic potential compared to either gene alone.

**Figure 4:**
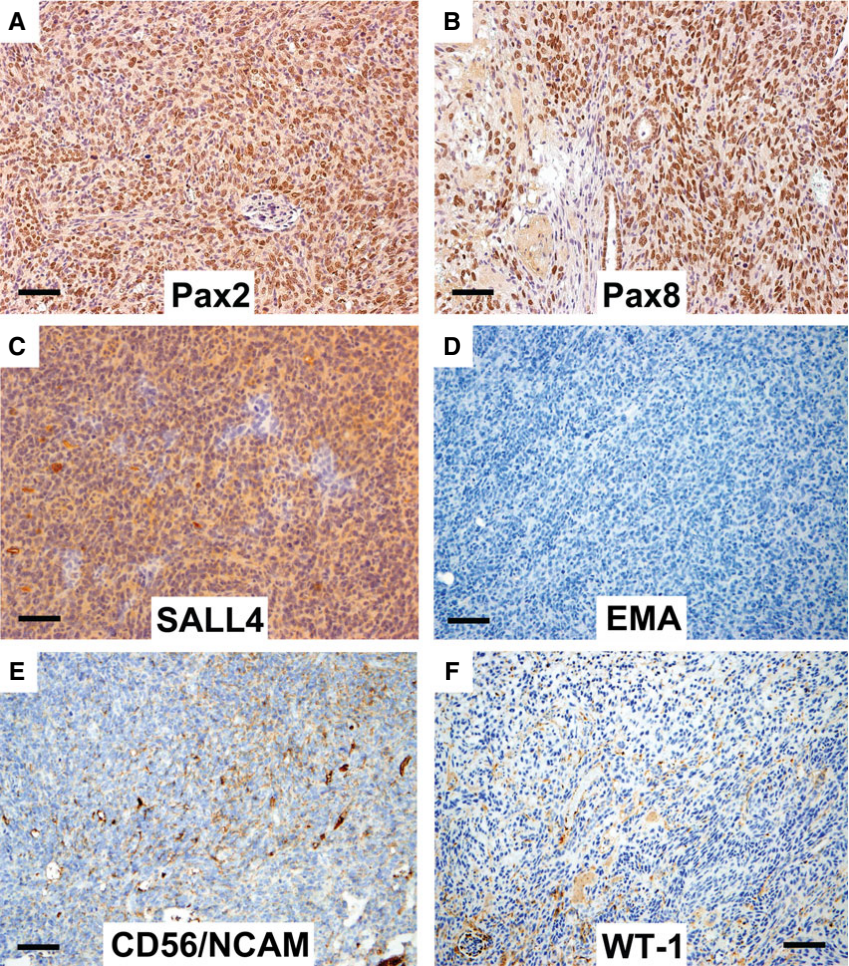
Tumors from orthotopically grafted Kras/Catnb cells show staining consistent with the epithelial component of human WT: The tumors from orthotopically grafted Kras/Catnb cells were stained for the following well‐defined markers of WT: Pax‐2 (A), Pax‐8 (B), SALL4 (C), EMA (D), CD56/NCAM (E), and WT1 (F). Bar represents 50 μm.

To verify that tumors from Kras/Catnb cells were consistent with WT, we stained the primary tumors with markers used to differentiate WT from other renal neoplasms. Consistent with our transgenic mouse model and WT patient cohort, the tumors stained for PAX8, PAX2, and weakly for SALL4 (Fig. 4A–C), while they did not stain for EMA and S‐100 (Fig. 4D and data not shown). As was the case in transgenic murine renal tumors, the grafted tumors did not stain for WT‐1 or CD56/NCAM (Fig. 4E,F). In summary, we developed a novel WT cell line model system that recapitulates our transgenic studies that can be used to study the mechanisms by which Kras drives metastatic disease progression in the presence of activated β‐catenin.

**Figure 5:**
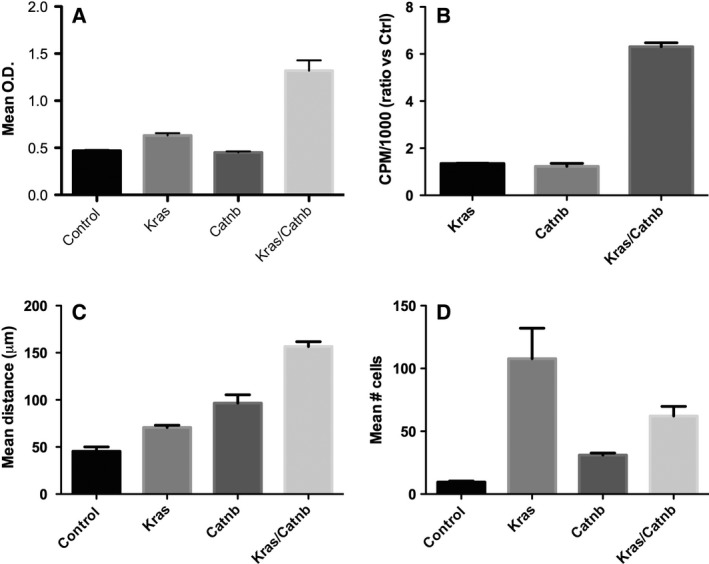
Coordinate activation of Ras and β‐catenin increases cellular growth and migration. MTS assay showing increased cellular growth in 1% FBS for 72 h for Kras/Catnb cells compared to Kras, Catnb, and control cells (A). Cellular proliferation was higher in Kras/Catnb cells when measured by tritiated thymidine incorporation normalized to control cells after serum starvation overnight followed by exposure to 1% FBS for 6 h (B). Cellular migration/wound closure by scratch assay over 24 h was highest in Kras/Catnb cells (C). Cellular invasion through Matrigel over 24 h was highest in Kras cells, although high levels were also noted in Kras/Catnb cells (D). All experiments were completed in triplicate with representative results shown. In all studies, the difference between Kras/Catnb and other cell types was significant (*P* < 0.05) except for Kras/Catnb compared to Kras invasion, panel D, *P* = 0.08.

To determine which cellular processes were enhanced by Ras to drive tumor growth and metastatic progression, we compared critical features of oncogenesis in Kras, Catnb, and Kras/Catnb cells. Growth kinetics measured by MTS assay were markedly accelerated in Kras/Catnb cells when compared to cells with activation of Ras or β‐catenin alone (Fig. 5A). This increased growth rate was due to increased cell proliferation as measured by tritiated thymidine incorporation (Fig. 5B), with no appreciable differences found in apoptosis as measured by cleaved caspase‐3 and PARP cleavage (data not shown). Two key features of metastatic potential *in vitro* include cellular migration and invasion. We measured transwell invasion through Matrigel and found that cells with Ras activation demonstrated the highest invasive phenotype. Interestingly, this invasive capacity was lower in the presence of activated Ras and β‐catenin compared to β‐catenin alone (Fig. 5D). Conversely, in a scratch assay, activation of either Ras or β‐catenin increased cellular migration relative to controls, but this was highest in cells with simultaneous activation of both pathways (Fig. 5C). Taken together, these data suggest that Ras combined with β‐catenin activation increases tumor growth and metastatic disease progression through increasing cellular proliferation and migration.

### 3.3 PI3K/AKT plays an important role in Ras‐mediated increases in cell growth and migration

**Figure 6:**
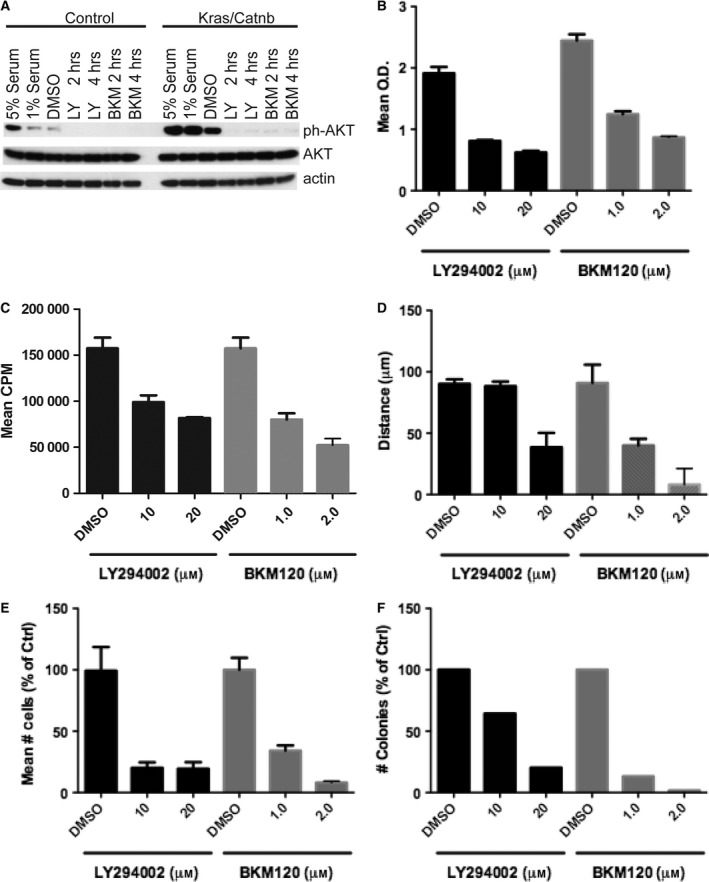
High levels of activated AKT in Kras/Catnb cells contribute to cellular growth, migration, invasion, and colony formation. Immunoblot shows high levels of p‐AKT in Kras/Catnb cells maintained in 5% or 1% FBS compared to controls that can be blocked by treatment with the PI3K inhibitors LY94002 or BKM120 (A). Both inhibitors demonstrated dose‐dependent inhibition of cellular growth by MTS assay (B), tritiated thymidine incorporation (C), cellular migration/wound closure (D), cellular invasion (E), and colony formation in soft agar in Kras/Catnb cells. All experiments were performed in 1% FBS and repeated in triplicate with representative results shown.

**Figure 7:**
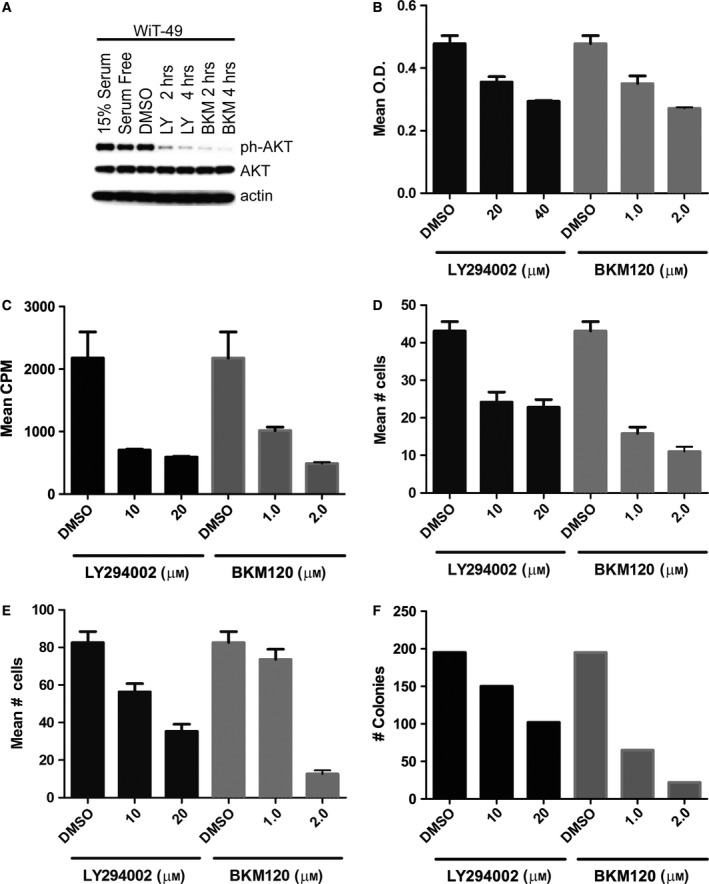
High levels of activated AKT in WiT49 cells contribute to cellular growth, migration, invasion, and colony formation. Immunoblot shows high levels of p‐AKT in WiT49 cells maintained in 5% or 1% FBS compared to controls that can be blocked by treatment with the PI3K inhibitors LY94002 and BKM120 (A). Both inhibitors demonstrated dose‐dependent inhibition of cellular growth by MTS assay (B), tritiated thymidine incorporation (C), cellular migration/wound closure (D), cellular invasion (E), and colony formation in soft agar in WiT49 cells. All experiments were performed in 1% FBS and repeated in triplicate with representative results shown.

As human WT have high levels of phosphorylated AKT, we tested whether cellular proliferation, migration, and transformation were dependent on the PI3K/AKT pathway. As in our transgenic model and human WT, Kras/Catnb cells had high levels of phosphorylated AKT levels when compared to controls (see Fig. 6A). To test whether the oncogenic features of Kras/Catnb cells were dependent on PI3K/AKT, we utilized the pan‐PI3K inhibitors LY94002 and BKM120. BKM120 is an oral pan‐PI3K inhibitor shown to be well tolerated and to have efficacy in early‐phase clinical trials (Ando *et al*., 2014; Bendell *et al*., 2012; Hyman *et al*., 2015; Rodon *et al*., 2014). Both PI3K inhibitors decreased cell growth/proliferation by MTS assay (Fig. 6B) and tritiated thymidine incorporation (Fig. 6C). Treatment with these inhibitors also decreased the migration of Kras/Catnb cells measured by scratch assay (Fig. 6D). Both cellular invasion through Matrigel (Fig. 6E) and colony formation in soft agar (Fig. 6F) of Kras/Catnb cells were inhibited by the PI3K inhibitors. We confirmed these results using a human WT cell line (WiT49), demonstrating inhibition of PI3K/AKT activation with both LY94002 and BKM120 (Fig. 7A) that inhibits cellular growth, proliferation, migration, invasion, and colony formation (Fig. 7B–F). These results strongly suggest that the transformed phenotype of renal epithelial cells, including human WT, depends in part on aberrant activation of the PI3K/AKT pathway.

### 3.4 Ras‐dependent metastatic disease progression is PI3K/AKT dependent

**Figure 8:**
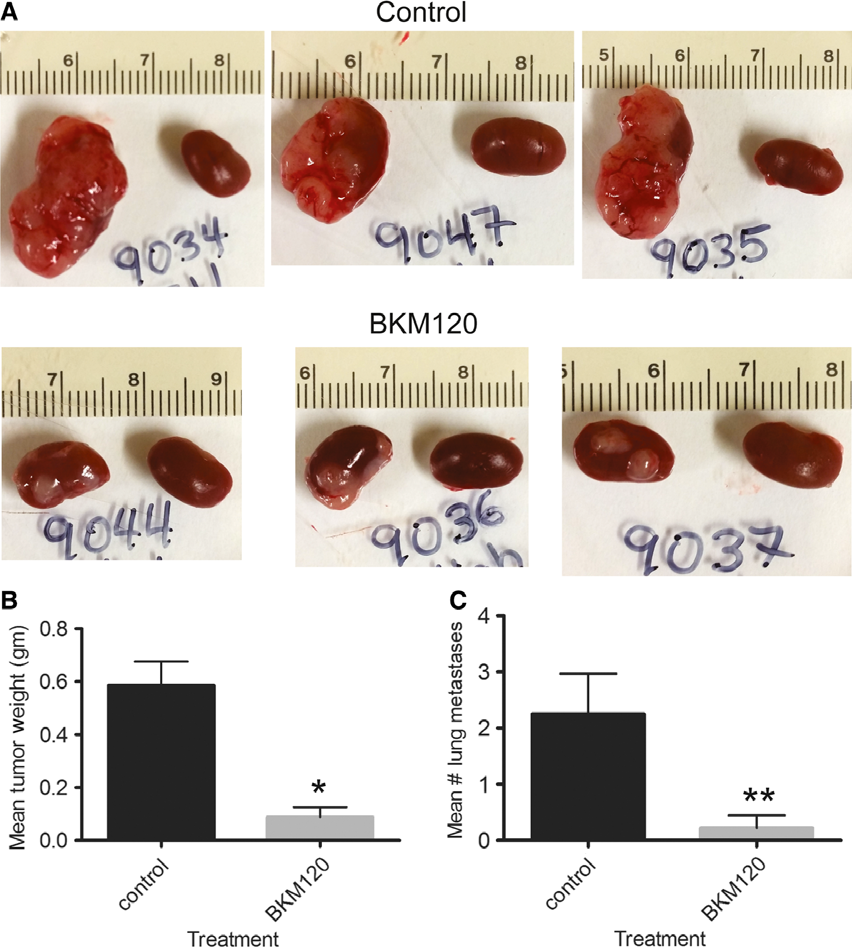
PI3K inhibition inhibits *in vivo* Kras/Catnb orthotopic tumor growth and metastatic progression. Kras/Catnb cells were grafted orthotopically in the subrenal capsule and allowed to grow for 2 weeks. Animals were then treated by oral gavage with 60 mg·kg^−1^·day^−1^ of BKM120 or vehicle control, three times per week, for 4 weeks. Animals were euthanized and the kidneys and lungs harvested, weighed, and fixed in formalin. Grossly mice treated with BKM120 had smaller tumor grafts than those treated with vehicle control (A). The tumor weight, expressed as the grafted kidney weight normalized to the contralateral control kidney, was markedly lower in animals treated with BKM120 (B, *P* = 0.0003). Inspection of the lungs showed metastases in one (11%) of nine BKM120‐treated mice compared to nine (75%, *P* = 0.0075) of 12 controls. The mean number of lung metastases per mouse in vehicle‐treated controls was 2.25 versus 0.22 in the BKM treatment arm (C, *P* = 0.0079).

To test whether inhibition of PI3K/AKT suppresses tumor growth *in vivo* and metastatic disease progression, we grafted Kras/Catnb renal epithelial cells under the renal capsule of 24 nude mice. After 2 weeks, the mice were divided into equal groups of 12 and treated with BKM120 or vehicle via oral gavage for 4 weeks, after which the animals were sacrificed and the tissues analyzed. One mouse in the BKM‐treated group died from complications directly related to the oral gavage. Two other mice in the BKM treatment arm died during therapy of unknown causes, although necropsy failed to demonstrate any gross tumor in either kidney. Consistent with the *in vitro* data, BKM120‐treated mice had significantly smaller renal tumors than vehicle‐treated controls (Fig. 8A,B, *P* = 0.0003). Inspection of the lungs by H&E and IHC for large T antigen demonstrated metastatic lesions in only one (11%) of nine surviving mice in the BKM treatment arm compared to nine of 12 (75%, *P* = 0.0075) in the control arm. In addition, for vehicle‐treated controls, the mean number of lung metastases per mouse was 2.25 in the control arm versus 0.22 in the BKM treatment arm (Fig. 8C, *P* = 0.0079).

## 4 Discussion

Only a third of WT harbor somatic mutations that can explain their increased canonical Wnt/β‐catenin activation. This suggests that other pathways play a critical role in WT biology. We previously showed β‐catenin activation is sufficient to induce murine renal tumors, but rapid tumor growth and metastatic disease progression require coordinate activation of Ras. We show herein that the same *KRAS* ^G12D^ mutations occur in human WT. Our murine model shows high levels of AKT activation, which is also present in the majority of human WT. When Ras and β‐catenin activations are combined, they markedly accelerate cellular proliferation, migration, and transformation *in vitro* and promote metastatic disease progression of orthotopically grafted renal epithelial cells. Cellular growth, transformation, and metastatic tumor progression are in part AKT dependent and are inhibited by pharmacological inhibition of PI3K/AKT. Thus, our study defines a potential role for *KRAS* mutations and PI3K/AKT pathway activation in human WT, which together represent candidate targets for therapy.

Ras pathway activation has been demonstrated in WT. However, until recently, it was presumed to be secondary to upstream up‐regulation of the IGF2 axis or mutations in genes such as SIX1/2, DGCR8, or DROSHA (Hu *et al*., 2011; Walz *et al*., 2015). These older studies failed to identify mutations in Ras family members using single‐strand conformation polymorphism analysis and direct DNA sequence analysis (Waber *et al*., 1993). Recently, our group found indirect evidence for *KRAS* mutations in 9‐32% of WT from Kenya using an Affymetrix‐based OncoScan™ (Lovvorn *et al*., 2015). We now show direct PCR evidence of functionally relevant *KRAS* ^G12D^ mutations in human WT. Taken together, these data suggest that RAS pathway activation is important in a subset of patients with WT.

Our study demonstrates evidence of AKT activation in the majority of human WT regardless of tumor histology. Further, we show that inhibition of PI3K/AKT can abrogate WT cellular growth, migration, and transformation *in vitro* and tumor growth and metastatic progression *in vivo*. A role for several receptor tyrosine kinases (RTKs), in particular the IGF/IGFR axis, has been demonstrated in WT. It is unclear whether these pathways signal through RAS, and if so, which downstream pathways are involved. One prior study showed activation of ERK1/2 in human WT samples, but the role of ERK in WT biology was not explored further (Hu *et al*., 2011). Another study demonstrated activation of the PI3K/AKT and MAPK/ERK pathways in WT cells due to increased IGF/IGFR signaling axis; however, the dependence of cellular growth and survival on either AKT or ERK was not tested (Bielen *et al*., 2012). Similarly, a study of serially propagated WT cells from xenografts in immunocompromised mice found evidence for PI3K/AKT activation in the NCAM+ cell subset, but again dependence on this pathway's activation was not explored (Pode‐Shakked *et al*., 2013). There has also been a study of one WT patient demonstrating activation of AKT by IHC, but a larger cohort was not examined (Subbiah *et al*., 2013). Herein, we demonstrated activation of PI3K/AKT in most human WT as well as a key role for this pathway in tumor growth and metastatic disease progression. This suggests the PI3K/AKT axis is a novel therapeutic target for WT.

An ongoing challenge in WT research is it to identify novel therapeutics to decrease the morbidity of current multimodality therapy and improve outcomes in children who relapse or become refractory to first‐line chemotherapy. Recently, there has been increasing interest in the PI3K/AKT pathway as a drug targetable pathway across several malignancies including breast and ovarian cancer (Mayer and Arteaga, 2016). Several compounds have now been developed, including the pan‐PI3K inhibitor BKM120 that is well tolerated and has promising efficacy in early‐phase clinical trials (Ando *et al*., 2014; Bedard *et al*., 2015; Bendell *et al*., 2012; Hyman *et al*., 2015; Mayer *et al*., 2014; Rodon *et al*., 2014). Our findings of a role for AKT activation in WT biology lays the foundation for exploring the utility of compounds like BKM120 or other inhibitors of the PI3K/AKT pathway in human WT.

In summary, we show that coordinate activation of Ras and β‐catenin drives AKT‐dependent tumor cell proliferation, migration, and colony formation, as well as tumor growth and lung metastases. In colon cancer, a mechanism by which RAS accelerates tumorigenesis is via AKT‐mediated modulation of β‐catenin's degradation, and increasing levels of cytosolic β‐catenin and canonical WNT/β‐catenin pathway activation. However, AKT activation can also amplify canonical WNT/β‐catenin pathway activation through phosphorylation of β‐catenin's C terminus, stabilizing the protein and enhancing TCF‐mediated transcription (Fang *et al*., 2007; Hino *et al*., 2005). There is also evidence that ERK can alter the transcriptional regulation of β‐catenin mRNA (Gosens *et al*., 2010). Therefore, we have shown activating *KRAS* mutations and a role for AKT in human WT. More work is required to define the mechanism by which AKT cooperates with β‐catenin to drive WT progression. These studies have important implications for developing novel therapeutic approaches to this disease.

## Author contributions

PEC conceived and designed the project and wrote the manuscript. RZ helped with project design and edited manuscript. DP and HDL designed and performed individual experiments. HLM and CLA helped with project design. HNL involved in study design and TMA construction and edited manuscript. HC interpreted IHC of TMA slides. ZS, KBD, and WP designed and performed SNaPshot experiments.

## Supporting information


**Fig. S1**. PCR confirmation of recombination in Kras, Catnb, and Kras/Catnb cell lines.

**Table S1** The SNaPshot screen queries 62 point mutations in 7 genes.
**Table S2.** SNaPshot screen multiplex‐PCR primers.
**Table S3.** Single‐base extension primers for the SNaPshot screen.
**Table S4.** SNaPshot screen spiking primers used for pan‐positive control assay.
