# Corrigendum to: Functional *KRAS* mutations and a potential role for PI3K/AKT activation in Wilms tumors

**DOI:** 10.1002/1878-0261.12453

**Published:** 2019-02-07

The following disclosure has been added to the original article (Polosukhina *et al*., 2017). Dr. Arteaga was on a Novartis Advisory Board at the time of publication, but the Board service was unrelated to this study.

## References

[mol212453-bib-0001] Polosukhina D , Love HD , Correa H , Su Z , Dahlman KB , Pao W , Moses HL , Arteaga CL , Lovvorn HN , Zent R *et al* (2017) Functional *KRAS* mutations and a potential role for PI3K/AKT activation in Wilms tumors. Mol Oncol 11, 405–421. 10.1002/1878-0261.12044 28188683 PMC5378659

