# Supplementary figures and images for: Functional KRAS mutations and a potential role for PI3K/AKT activation in Wilms tumors
Source: Mol Oncol. 2017 Mar 15;11(4):405–21. doi: 10.1002/1878-0261.12044 (PMC5378659; doi:10.1002/1878-0261.12044)

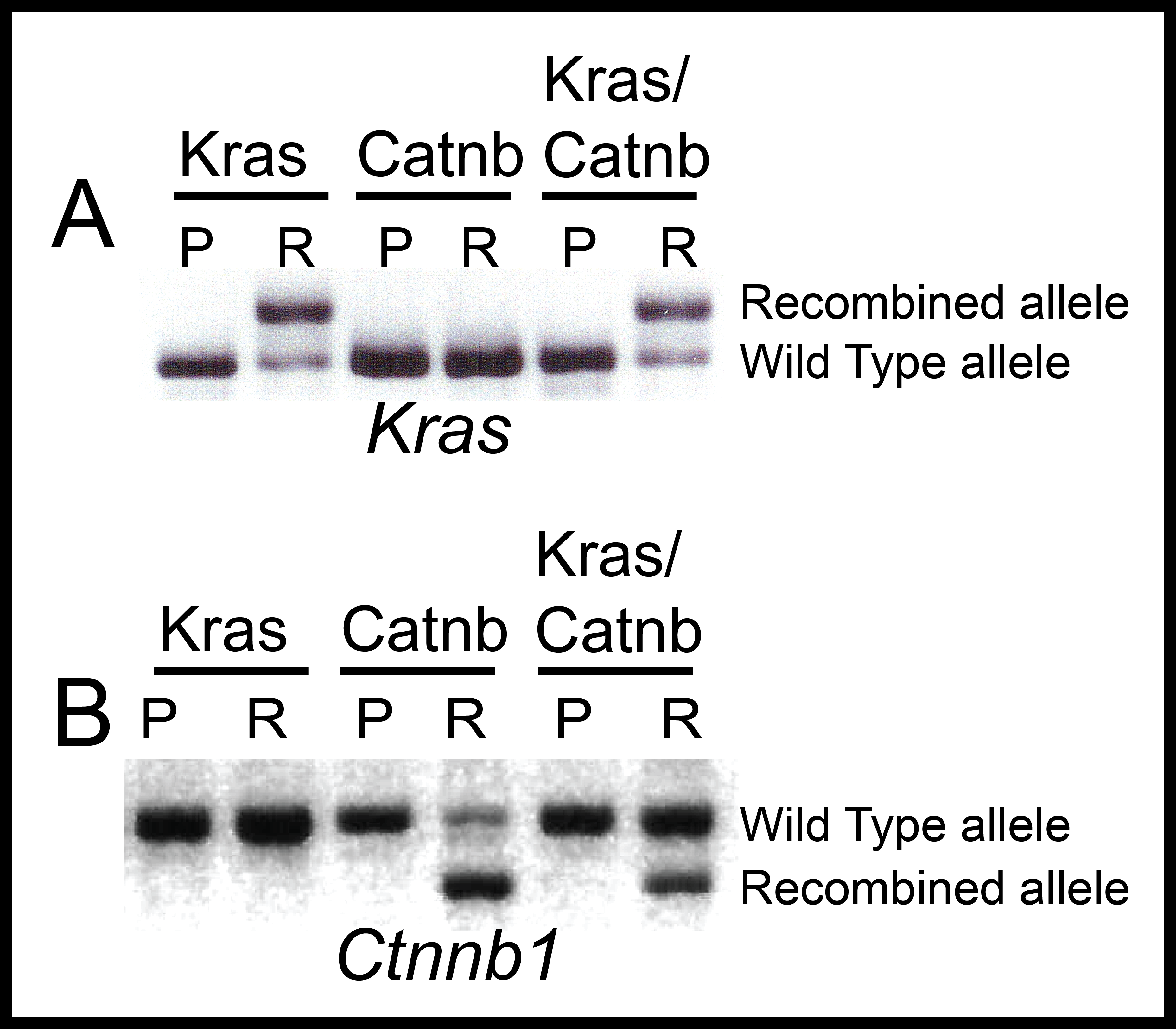

Supplement: Supplementary file 1 — Fig. S1. PCR confirmation of recombination in Kras, Catnb, and Kras/Catnb cell lines. [file MOL2-11-405-s001.tif]
