# Supplementary material for: Functional KRAS mutations and a potential role for PI3K/AKT activation in Wilms tumors
Source: Mol Oncol. 2017 Mar 15;11(4):405–21. doi: 10.1002/1878-0261.12044 (PMC5378659; doi:10.1002/1878-0261.12044)
Supplement: Supplementary file 2 — Table S1 The SNaPshot screen queries 62 point mutations in 7 genes. Table S2. SNaPshot screen multiplex‐PCR primers. Table S3. Single‐base extension primers for the SNaPshot screen. Table S4. SNaPshot screen spiking primers used for pan‐positive control assay. [file MOL2-11-405-s002.doc]

Supplementary Table 1. The SNaPshot screen queries 62 point mutations in 7 genes.

| ***KRAS*** |  |  |  | ***PIK3CA*** |  |  |
| --- | --- | --- | --- | --- | --- | --- |
| **Position** | **AA mutant** | **Nucleotide mutant** |  | **Position** | **AA mutant** | **Nucleotide mutant** |
| G12 | p.G12C | c.34G>T |  | H1047 | p.H1047R | c.3140A>G |
|  | p.G12S | c.34G>A |  |  | p.H1047L | c.3140A>T |
|  | p.G12R | c.34G>C |  | E542 | p.E542K | c.1624G>A |
|  | p.G12V | c.35G>T |  | E545 | p.E545K | c.1633G>A |
|  | p.G12A | c.35G>C |  |  | p.E545Q | c.1633G>C |
|  | p.G12D | c.35G>A |  |  | p.E545A | c.1634A>C |
| G13 | p.G13C | c.37G>T |  |  | p.E545G | c.1634A>G |
|  | p.G13S | c.37G>A |  |  | p.E545V | c.1634A>T |
|  | p.G13R | c.37G>C |  | Q546 | p.Q546K | c.1636C>A |
|  | p.G13D | c.38G>A |  |  | p.Q546E | c.1636C>G |
|  | p.G13A | c.38G>C |  |  | p.Q546P | c.1637A>C |
|  | p.G13V | c.38G>T |  |  | p.Q546R | c.1637A>G |
| Q61 | p.Q61K | c.181C>A |  |  | p.Q546L | c.1637A>T |
|  | p.Q61R | c.182A>G |  | D549 | p.D549N | c.1645G>A |
|  | p.Q61L | c.182A>T |  |  |  |  |
|  | p.Q61H | c.183A>T |  | ***SMAD4*** |  |  |
|  |  | c.183A>C |  | E330 | p.E330A | c.989A>C |
| A146 | p.A146T | c.436G>A |  | D351 | p.D351H | c.1051G>C |
|  | p.A146P | c.436G>C |  |  | p.D351N | c.1051G>A |
|  | p.A146V | c.437C>T |  | D355 | p.D355E | c.1065C>A |
| K117 | p.K117N | c.351A>C |  | R361 | p.R361S | c.1081C>A |
|  |  | c.351A>T |  |  | p.R361C | c.1081C>T |
|  |  |  |  |  | p.R361H | c.1082G>A |
| ***BRAF*** |  |  |  |  |  |  |
| G466 | p.G466V | c.1397G>T |  | ***PTEN*** |  |  |
| G469 | p.G469E | c.1406G>A |  | R233 | p.R233* | c.697C>T |
|  | p.G469A | c.1406G>C |  | R159 | p.R159S | c.477G>T |
|  | p.G469V | c.1406G>T |  | R267 | p.R267fs*9 | c.800delA |
| D594 | p.D594G | c.1781A>G |  |  |  |  |
|  | p.D594V | c.1781A>T |  | ***NRAS*** |  |  |
| G596 | p.G596R | c.1786G>C |  | G12 | p.G12C | c.34G>T |
| V600 | p.V600E | c.1799T>A |  |  | p.G12S | c.34G>A |
|  |  |  |  |  | p.G12A | c.35G>C |
| ***AKT*** |  |  |  |  | p.G12D | c.35G>A |
| E17 | p.E17K | c.49G>A |  |  | p.G12V | c.35G>T |
|  |  |  |  | Q61 | p.Q61K | c.181C>A |
|  |  |  |  |  | p.Q61R | c.182A>G |

*This mutation results in a premature stop codon

fs*9 This mutation results in a deletion-frameshift

| **Amplification primer name** | **Primer sequence^a^** | **Product length (bp)** |
| --- | --- | --- |
| AKT1_ex2_a1^b^ | GAGGGTCTGACGGGTAGAGT | 95 |
| AKT1_ex2_a2^b^ | TCTTGAGGAGGAAGTAGCGT |
| BRAF_ex11_a1^b^ | TCTGTTTGGCTTGACTTGACTT | 75 |
| BRAF_ex11_a2^b^ | TCACCACATTACATACTTACCATGC |
| BRAF_ex15_a1^b^ | TGCTTGCTCTGATAGGAAAATG | 143 |
| BRAF_ex15_a2^b^ | CTGATGGGACCCACTCCAT |
| KRAS_ex2_a1^b^ | TCATTATTTTTATTATAAGGCCTGCTG | 186 |
| KRAS_ex2_a2^b^ | AGAATGGTCCTGCACCAGTAA |
| KRAS_ex3_a1^b^ | AATTGATGGAGAAACCTGTCTCTTG | 75 |
| KRAS_ex3_a2^b^ | TGGTCCCTCATTGCACTGTA |
| KRAS_ex4_a1 | AGAGTTAAGGACTCTGAAGATGTAC | 152 |
| KRAS_ex4_a2 | CTTACCTGTCTTGTCTTTGC |
| NRAS_ex2_a1 | CAACAGGTTCTTGCTGGTGT | 175 |
| NRAS_ex2_a2 | GAGAGACAGGATCAGGTCAGC |
| NRAS_ex3_a1^b^ | ATAGATGGTGAAACCTGTTTGTTGG | 79 |
| NRAS_ex2_a2^b^ | TGTATTGGTCTCTCATGGCACT |
| PIK3CA_ex9_a1^b^ | GACAAAGAACAGCTCAAAGCAA | 98 |
| PIK3CA_ex9_a2^b^ | TTTAGCACTTACCTGTGACTCCA |
| PIK3CA_ex20_a1^b^ | GAGCAAGAGGCTTTGGAGTA | 80 |
| PIK3CA_ex20_a2^b^ | ATCCAATCCATTTTTGTTGTCC |
| PTEN_ex5_b1 | GGGCAAATTTTTAAAGGCACAA | 106 |
| PTEN_ex5_b2 | CCAGGAAGAGGAAAGGAAAAA |
| PTEN_ex7_a1^b^ | GGTGAAGATATATTCCTCCAATTCA | 123 |
| PTEN_ex7_a2^b^ | TTCTCCCAATGAAAGTAAAGTACAAA |
| SMAD4_exon 8_a1 | TTTCTCATGGGAGGATGTTCTTTCC | 103 |
| SMAD4_exon 8_a2 | TGTCTCTCCTACCTGAACATCCA |
| SMAD4_exon 8_b1 | GGTTCCTTCAAGCTGCCCTATTGT | 107 |
| SMAD4_exon 8_b2 | TGGCTTCTGTCCTGTGGACATTGG |

Supplementary Table 2. SNaPshot screen multiplex-PCR primers.

^a^The sequences are shown 5’>3’.

^b^Primer sequences were published previously .

Supplementary Table 3. Single-base extension primers for the SNaPshot screen.

| **Extension primer name^a^** | **Primer sequence^b^** | **Primer length (nucleotides)** |
| --- | --- | --- |
| AKT149_extR^c^ | **ACTGACTGACTGACTGACTGACTGACTGACTGACTGACTGACTGACTGACT**CGCCAGGTCTTGATGTACT | 70 |
| BRAF1781_extF | **GACTGACTGACTGACTGACTGACTGACTGACT**CCTCACAGTAAAAATAGGTG | 52 |
| BRAF1786_extF | **GACTGACTGACTGACTGACTGACTGACTGACTGACTGACTGACTGACTGACTGACTGACTGACTGACT**CACAGTAAAAATAGGTGATTTT | 90 |
| BRAF1397_extF^c^ | **TGACTGACTGACTGACTGACTGACTGACTGACTGACTGACTGACTGACTGACTGAC**GGGACAAAGAATTGGATCTG | 76 |
| BRAF1406_extF^c^ | **AGACTGACTGACTGACTGACTGACTGACTGACTG**GAATTGGATCTGGATCATTTG | 55 |
| BRAF1799_extF^c^ | **GACTGACTGACTGACTGACTGACT**GTGATTTTGGTCTAGCTACAG | 45 |
| KRAS34_extR^c^ | **GACTGACTG**CTCTTGCCTACGCCAC | 25 |
| KRAS351_extR | **GACTGACTGACTGACTGACT**GTTCTAGAAGGCAAATCACA | 40 |
| KRAS436_extF | **GACTGACTGACTGACTGACTGACT**GAATTCCTTTTATTGAAACATCA | 47 |
| KRAS437_extF | GACTGACTGACTGACTGACTGACTGACTGACTGACTGACTGACTGACTGACTGACTATTCCTTTTATTGAAACATCAG | 78 |
| KRAS181_extF^c^ | **CTGACTGACTGACTGACTGACTGACTGACTGACTGACTGACTGACTGACTGACTGACT**ATTCTCGACACAGCAGGT | 76 |
| KRAS182_extF^c^ | **GACTGACTGACTGACTGACT**ATTCTCGACACAGCAGGTC | 39 |
| KRAS183_extR^c^ | **ACTGACTGACTGACTGACTGACTGACTGACTGACTGACTGACTGAC**CTCATTGCACTGTACTCCTC | 66 |
| KRAS35_extF^c^ | **CTGACT**CTTGTGGTAGTTGGAGCTG | 25 |
| KRAS37_extF^c^ | **TGACTGACTGA**TGGTAGTTGGAGCTGGT | 28 |
| KRAS38_extF^c^ | **GACTGACTGAC**GGTAGTTGGAGCTGGTG | 28 |
| NRAS34_extR | GCTTTTCCCAACACCAC | 17 |
| NRAS35_extR | CGCTTTTCCCAACACCA | 17 |
| NRAS181_extF^c^ | **GACTGACTGACTGACTGACTGACTGACTGACTGAC**ACATACTGGATACAGCTGGA | 55 |
| NRAS182_extF^c^ | **CTGACTGACTGACTGACTGACTGACTGACTGACTG**CATACTGGATACAGCTGGAC | 55 |
| PIK3CA1633_extF^c^ | **GACTGACTGACTGACTGACTGACTGACTGACTGACTGACTGACT**GATCCTCTCTCTGAAATCACT | 65 |
| PIK3CA1624_extR^c^ | **TGACTGACTGACTGACTGACTGACTGACTGACTGACTGACTGAC**TTCTCCTGCTCAGTGATTT | 63 |
| PI3K1636_extF | **GACTGACTGACTGACTGACTGACTGACTGACTGACTGACTGACT**CCTCTCTCTGAAATCACTGAG | 65 |
| PI3K1637_extF | **GACTGACTGACTGACTGACTGACTGACTGACTGACTGAC**CTCTCTCTGAAATCACTGAGC | 60 |
| PIK3CA3140_extF | **GACTGACTGACT**TTTCATGAAACAAATGAATGATGCAC | 38 |
| PIK3CA1645_extR | **GACTGACTGACTGACTGACTGACTGACTGACTGACTGACTGACTGACTGACT**CCTGTGACTCCATAGAAAAT | 72 |
| PIK3CA1634_extF | CTCTCTCTGAAATCACTG | 18 |
| PTEN477_extF | **GACTGACTGACTGACTGACTGACTGACTGACTGACTGACTGACTGACTGACTGACTGACTGACT**GATTTCTATGGGGAAGTAAG | 84 |
| PTEN697_extF | **ACTGACTGACTGACTGACTGACTGACTGACTGACTGA**CCAATTCAGGACCCACA | 54 |
| PTEN800delA_extF | **GACTGACTGACTGACTGACTGACTGACTGACTGACTGACT**AAACAGAACAAGATGCTAAAAA | 62 |
| SMAD4989_extF | **GACTGACTGACTGACT**GTGTTCCATTGCTTACTTTG | 36 |
| SMAD41051_extF | **GACTGACTGACTGACTGACTGACTGACTGACTGACTGACTGACTGACTGACTGACTGACTGACT**GCTGCCCTATTGTTACTGTT | 84 |
| SMAD41065_extR | **GACTGAGACTGACTGACTGACTGACTGACTGACTGACTGACTGACTGACTGACTGACT**GCGATCTCCTCCAGAAGG | 76 |
| SMAD41081_extR | **GACTGACTGACTGACTGACTGACTGA**GAGTTGACCCAAACAAAAGC | 46 |
| SMAD41082_extR | **GACTGACTGACTGACTGACTGACTGACTGACTGACTGACTGACTGACTGACT**GAGTTGACCCAAACAAAAG | 71 |

^a^Primers were purified by polyacrylamide gel electrophoresis.

^b^The sequences are shown 5’>3’ and bold nucleotides are repetitive GACT sequence used to adjust product size.

^c^Primer sequences were published previously .

Supplementary Table 4. SNaPshot screen spiking primers used for pan-positive control assay.

| **Spiking primer name** | **Primer sequence^a^** |
| --- | --- |
| KRAS34G>A(R)^b^ | GTTGGAGCTAGTGGCGTAGGCAAGAGTGCCAAAAA |
| KRAS34G>C(R)^b^ | GTTGGAGCTCGTGGCGTAGGCAAGAGTGCCAAAAA |
| KRAS34G>T(R)^b^ | GTTGGAGCTTGTGGCGTAGGCAAGAGTGCCAAAAA |
| SMAD989A>C | ACATCCATTGCAAAGTAAGCAATGGAACACAAAAA |
| KRAS436G>A | TTGTCTTTGTTGATGTTTCAATAAAAGGAATTAAA |
| KRAS436G>C | TTGTCTTTGGTGATGTTTCAATAAAAGGAATTAAA |
| NRAS181C>A^b^ | ACTCTTCTTTTCCAGCTGTATCCAGTATGTAAAAA |
| PIK3CA1633G>A^b^ | CTCCTGCTTAGTGATTTCAGAGAGAGGATCAAAAA |
| PIK3CA1633G>C^b^ | CTCCTGCTGAGTGATTTCAGAGAGAGGATCAAAAA |
| BRAF1397G>T^b^ | CCAAATGATACAGATCCAATTCTTTGTCCCAAAAA |
| PTEN477G>T | GTCTCTGGTACTTACTTCCCCATAGAAATCAAAAA |
| BRAF1786G>C | TAGCTAGACGAAAATCACCTATTTTTACTGAAAAA |
| PIK3CA1634A>C | TCTCCTGCGCAGTGATTTCAGAGAGAGGATAAAA |
| PIK3CA1634A>G | TCTCCTGCCCAGTGATTTCAGAGAGAGGATAAAA |
| PIK3CA1634A>T | TCTCCTGCACAGTGATTTCAGAGAGAGGATAAAA |
| KRAS38G>A^b^ | TTGCCTACGTCACCAGCTCCAACTACCACAAAAAA |
| KRAS38G>C^b^ | TTGCCTACGGCACCAGCTCCAACTACCACAAAAAA |
| KRAS38G>T | TTGCCTACGACACCAGCTCCAACTACCACAAAAAA |
| BRAF1799T>A^b^ | CGAGATTTCTCTGTAGCTAGACCAAAATCAAAAAA |
| NRAS182A>G^b^ | ACTCTTCTCGTCCAGCTGTATCCAGTATGTAAAAA |
| PTEN800A(G) | GTACAAACCTTTTTAGCATCTTGTTCTGTAAAAA |
| AKT49G>A(R)^b^ | CTGTAGGGAAGTACATCAAGACCTGGCGGCAAAAA |
| KRAS437C>T | TTGTCTTTACTGATGTTTCAATAAAAGGAATTAAA |
| SMAD1051G>C | CGTATCCATGAACAGTAACAATAGGGCAGCAAAAA |
| SMAD1051G>A | CGTATCCATTAACAGTAACAATAGGGCAGCAAAAA |
| KRAS35G>A^b^ | GCCTACGCCATCAGCTCCAACTACCACAAGTTAAA |
| KRAS35G>C^b^ | GCCTACGCCAGCAGCTCCAACTACCACAAGTTAAA |
| KRAS35G>T ^b^ | GCCTACGCCAACAGCTCCAACTACCACAAGTTAAA |
| KRAS182A>G^b^ | TACTCCTCTCGACCTGCTGTGTCGAGAATAAAAAA |
| KRAS182A>T^b^ | TACTCCTCTAGACCTGCTGTGTCGAGAATAAAAAA |
| BRAF1781A>G | AGACCAAAACCACCTATTTTTACTGTGAGGTAAAA |
| BRAF1781A>T | AGACCAAAAACACCTATTTTTACTGTGAGGTAAAA |
| PIK3CA1624G>A(R)^b^ | TCCTCTCTCTAAAATCACTGAGCAGGAGAAAGAAA |
| SMAD1065C>A(R) | ATACGTGGAACCTTCTGGAGGAGATCGCTTAAAAA |
| NRAS34G>A(R) | ACTGGTGGTGGTTGGAGCAAGTGGTGTTGGGAAAAGCGCAAAAAA |
| NRAS34G>C(R)^c^ | ACTGGTGGTGGTTGGAGCACGTGGTGTTGGGAAAAGCGCAAAAA |
| NRAS34G>T(R)^c^ | ACTGGTGGTGGTTGGAGCATGTGGTGTTGGGAAAAGCGCAAAAA |
| PIK3CA3140A>G^b^ | TGACGTGCATCATTCATTTGTTTCATGAAAAAAAA |
| PIK3CA3140A>T | TGAAGTGCATCATTCATTTGTTTCATGAAAAAAAA |
| SMAD1081C>A(R) | GGAGGAGATAGCTTTTGTTTGGGTCAACTCTCAAAA |
| SMAD1081C>T(R) | GGAGGAGATTGCTTTTGTTTGGGTCAACTCTCAAAA |
| PTEN697C>T (F) | TTCCCGTCATGTGGGTCCTGAATTGGAGGAATAAAA |
| PIK3CA1637A>C | CTTTCTCCGGCTCAGTGATTTCAGAGAGAGAAA |
| PIK3CA1637A>G | CTTTCTCCCGCTCAGTGATTTCAGAGAGAGAAA |
| PIK3CA1637A>T | CTTTCTCCAGCTCAGTGATTTCAGAGAGAGAAA |
| KRAS183A>C(R)^b^ | GCAGGTCACGAGGAGTACAGTGCAATGAGGAAAAA |
| KRAS183A>T(R)^b^ | GCAGGTCATGAGGAGTACAGTGCAATGAGGAAAAA |
| PIK3CA1645G>A(R) | CAGGAGAAAAATTTTCTATGGAGTCACAGAAAAA |
| NRAS35G>A (R) | GTTGGAGCAGATGGTGTTGGGAAAAGCGCACAAAA |
| NRAS35G>T (R) | GTTGGAGCAGTTGGTGTTGGGAAAAGCGCACTAAAAA |
| KRAS37G>A^b^ | CCTACGCTACCAGCTCCAACTACCACAAGTAAAAA |
| KRAS37G>C^b^ | CCTACGCGACCAGCTCCAACTACCACAAGTAAAAA |
| KRAS37G>T^b^ | TTGCCTACGCAACCAGCTCCAACTACCACAAAAAA |
| KRAS351A>C (R) | GAAATAACTGTGATTTGCCTTCTAGAACAGAAAAA |
| KRAS351A>T (R) | GAAATAATTGTGATTTGCCTTCTAGAACAGAAAAA |
| BRAF1406G>A | GACTGTTTCAAATGATCCAGATCCAATTCTAAAAA |
| BRAF1406G>C^b^ | GACTGTTGCAAATGATCCAGATCCAATTCTAAAAA |
| BRAF1406G>T | GACTGTTACAAATGATCCAGATCCAATTCTAAAAA |
| PIK3CA1636C>A | CTTTCTCCTTCTCAGTGATTTCAGAGAGAGAAA |
| PIK3CA1636C>G | CTTTCTCCTCCTCAGTGATTTCAGAGAGAGAAA |
| SMAD1082G>A | GGAGGAGATCACTTTTGTTTGGGTCAACTCTCAAAA |
| KRAS181C>A^b^ | ACTCCTCTTTACCTGCTGTGTCGAGAATATAAAAA |

^a^The sequences are shown 5’>3’

^b^Primer sequences were published previously .

^c^Primer sequences were published previously .
